# The microbiome extends host evolutionary potential

**DOI:** 10.1038/s41467-021-25315-x

**Published:** 2021-08-26

**Authors:** Lucas P. Henry, Marjolein Bruijning, Simon K. G. Forsberg, Julien F. Ayroles

**Affiliations:** 1 Dept. of Ecology and Evolutionary Biology, Princeton University, Princeton, NJ USA; 2 Lewis-Sigler Institute for Integrative Genomics, Princeton University, Princeton, NJ USA; 3 Dept. of Medical Biochemistry and Microbiology, Uppsala University, Uppsala, Sweden

**Keywords:** Evolutionary ecology, Experimental evolution, Symbiosis, Microbiome

## Abstract

The microbiome shapes many host traits, yet the biology of microbiomes challenges traditional evolutionary models. Here, we illustrate how integrating the microbiome into quantitative genetics can help untangle complexities of host-microbiome evolution. We describe two general ways in which the microbiome may affect host evolutionary potential: by shifting the mean host phenotype and by changing the variance in host phenotype in the population. We synthesize the literature across diverse taxa and discuss how these scenarios could shape the host response to selection. We conclude by outlining key avenues of research to improve our understanding of the complex interplay between hosts and microbiomes.

## Abstract (web summary)

The microbiome is becoming recognized as a key determinant of host phenotype. Here, Henry et al. present a framework for building our understanding of how the microbiome also influences host evolution, review empirical examples and research approaches, and highlight emerging questions.

## Introduction

The “microbiome” has emerged as a key determinant of many aspects of organismal biology, capable of shaping developmental, physiological, and reproductive phenotypes^1–5^. Yet, the contribution of the microbiome to host adaptation remains an evolutionary puzzle^6–9^. Microbiomes are traditionally viewed as nongenetic, environmental factors that influence host phenotypes. However, unlike abiotic environmental conditions, the effects of microbial variation have a genetic basis and can evolve^10^, but are not always inherited in the same way as host genes^6,7,9^. While microbiomes have substantial phenotypic effects on their hosts, these effects strongly depend on the ecological context^2,3,8^. Despite their clear importance, the complex effects of microbial inheritance and genetics on host phenotypes remain underappreciated, limiting our ability to understand host-microbiome evolution.

In this perspective piece, we explore how the microbiome influences host phenotypic variation. If the microbiome expands the host genetic repertoire and influences trait heritability, then the microbiome may have substantial impacts on host phenotypic evolution. We propose a path forward by integrating the microbiome into quantitative genetic models. Models that account for patterns of microbial inheritance and phenotypic effects will allow researchers to make predictions into the microbial impact on host evolutionary trajectories. Using this perspective, we detail two common scenarios found in the literature. First, microbial variation may shift the mean phenotype of the population, as would be expected when hosts leverage the microbiome to become locally adapted. Second, microbial variation may change host phenotypic variance, which could reduce variance by buffering against host variation or increase variability within the population. These two patterns may occur together (e.g., shift in mean and decrease in variance) and will change how hosts explore their fitness landscape. We review approaches to measure host-microbiome evolution, addressing current theoretical and technical limitations. We conclude by suggesting key avenues of research powered by the integration of the microbiome into common evolutionary tools, like quantitative genetics and experimental evolution. Through this perspective piece, we highlight how considering shifts in phenotypic mean and variance will help elucidate how the microbiome influences host evolution.

### Phenotypic effects: extending the host genetic repertoire

Dawkins’ “*Extended Phenotype*” recognized how organisms modify surrounding environments and ecological communities^11,12^. Through environmental modification, an organism’s phenotypic effects are extended beyond its own genome, suggesting evolution is influenced through interacting ecological communities. This theory, developed for free-living ecosystems, also applies to host-microbiome interactions^12,13^. The microbiome, with its consortium of genomes, extends the genetic repertoire of the host to form what some are now calling the “*Extended Genotype*” because the host integrates the extended effects of the microbiome into its phenotype^3,14–22^. This extended genetic repertoire may shape the distribution of host phenotypes within a population, and consequently, shape the evolutionary potential of the host.

To formalize these verbal arguments, there is a genuine need for quantitative genetic models that incorporate the contribution of the microbiome to host phenotype, and subsequently, response to selection (Box 1). Explicitly incorporating the genetic variance encoded by microbes (V_G-MICRO_) as one would for the other components of phenotypic variance (e.g., *V*_G-HOST_ or *V*_E_) should be a useful starting point. Host phenotypic variance can then be decomposed as:

1 $${V}_{P}={V}_{{{{{{\rm{G-HOST}}}}}}}+{V}_{{{{{{\rm{G-MICRO}}}}}}}+{V}_{{{{{{\rm{E}}}}}}}$$

**Fig. 1:**
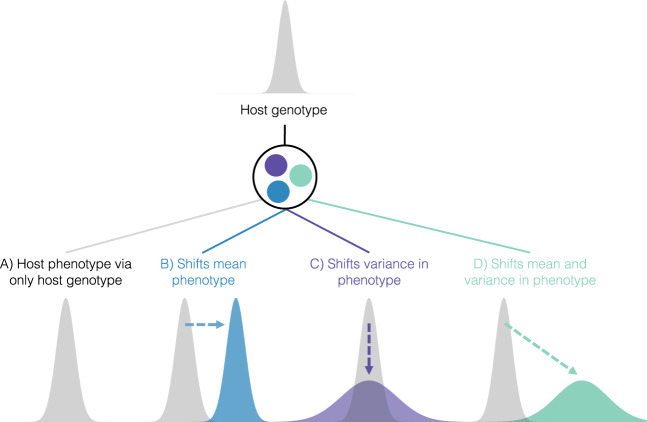
Microbial influence on host phenotypic variation. The microbiome encodes many more genes than the host genome alone. Interactions with variation in the microbiome may alter the host genotype-phenotype map, shaping host phenotypic variance within populations. **A** First, some host phenotypes will not be affected by the microbiome (visualized in gray) and it is worth noting that not all microbes will influence host phenotypes. **B** The microbiome may shift the mean host phenotypes. The blue distribution is suggestive of when hosts leverage locally adaptive microbiomes to match local selective pressures. **C** Alternatively, the microbiome may also alter phenotypic variance (conceptualized in purple). **D** Finally, both the phenotypic mean and variance may be affected by the microbiome (in green). We note here that these scenarios are not mutually exclusive. Expanding host phenotypic variation through the microbiome may allow hosts to explore novel regions of fitness landscapes. These are conceptualized phenotypic distributions, and more experimental work is necessary to confirm how the microbiome affects host phenotypic distributions.

When *V*_G-MICRO_ contributes to host phenotypic variance, the microbiome may shape the evolutionary potential for a population. For illustrative purposes, imagine a scenario where a microbe turns a host blue (i.e., *V*_G-MICRO_ contributes substantially to *V*_P_), and this blue phenotype increases host fitness in a hypothetical environment. We would predict that these beneficial blue-inducing microbes will increase in frequency in the host population, shifting the mean host color. We consider this scenario as hosts leveraging locally adaptive microbes (Fig. 1). The evolutionary benefits in this scenario will depend on the match between phenotype and selective pressure. Second, microbial genetic variation may alter variance in host phenotypes within a population (Fig. 1). The microbiome may effectively act as a buffer against environmental perturbation, thus decreasing phenotypic variance. Under this scenario, modifying the degree of host phenotypic robustness may be advantageous for specialization, but may constrain future adaptation if the environment is not constant^23,24^. On the other hand, the microbiome may also increase host phenotypic variance. This may enable the simultaneous exploration of novel regions of fitness landscapes, potentially enhancing host evolutionary responses to rapidly changing environments^25–28^. We note that these scenarios are not mutually exclusive, and the microbiome may shape both mean and variance in a population. Finally, it is important to consider that not all host phenotypes will be influenced by the microbiome, and not all microbes will influence host phenotypes (e.g., *V*_G-MICRO_ = 0). Naturally, many host-microbiome interactions are also pathogenic, but such relationships have been studied theoretically and empirically in disease ecology^8,29^ and could also be incorporated into our quantitative genetics framework. Here, however, we are primarily focusing on adaptive microbiomes.

Using this simple quantitative genetics framework enables the partitioning of the genetic contribution of the microbiome to host phenotypic variance, and subsequently, host evolution. We explore how the two broad scenarios of the microbiome on host phenotypic effects described above (shifting mean phenotype and changing variance) can affect host evolutionary potential (Box 1). Overall, when the microbiome contributes to host phenotypes, the response to selection is amplified and shifts host evolutionary trajectory. However, the magnitude of microbial effects on host evolution will also strongly depend on the complexities of microbial inheritance.

Box 1: Quantitative genetics of host–microbiome interactions Quantitative genetic models traditionally decompose host phenotypic variance into genetic (*V*_G_) and environmental (*V*_E_) components. However, this simple model generally does not consider the contribution of the microbiome to host phenotypic variation. Below, we provide the intuition for how a quantitative genetic framework that incorporates the microbiome could provide the quantitative foundation to study the contribution of the microbiome to host evolution. Ignoring the contribution of epistasis and dominance effects and assuming no covariances between host genetic, microbial, and environmental effects (a common starting point in quantitative genetics), the total phenotypic variance can be modeled as: *V*_*P*_ *=* *V*_*G-HOST*_+*V*_*G-MICRO*_+*V*_*E*_ Where *V*_G-HOST_ is the genetic variance in the host contributing to phenotype, *V*_G-MICRO_ is genetic variance in the microbiome contributing to host phenotype, and *V*_E_ is the environmental variance. *V*_G-MICRO_ is frequently quantified as the relative abundance of specific microbes that contribute to the host phenotypic variance. However, with this framework, *V*_G-MICRO_ can be extended to strain-level variation (e.g., SNPs within or between species in the microbiome). Recent studies of the human microbiome and in agricultural breeding studies^35,48,49,142–144^ suggest that, if we assume that all microbial genetic variance is faithfully transmitted from parent to offspring, host heritability can be calculated as: ${h}_{{{{{{\rm{TOTAL}}}}}}}^{2}={h}_{{{{{{\rm{HOST}}}}}}}^{2}+{h}_{{{{{{\rm{MICRO}}}}}}}^{2}=\frac{{V}_{{{{{{\rm{G}}}}}}-{{{{{\rm{HOST}}}}}}}+{V}_{{{{{{\rm{G}}}}}}-{{{{{\rm{MICRO}}}}}}}}{{V}_{{{{{{\rm{P}}}}}}}}$. This simple model conveys how the contribution of the microbiome to host phenotypic variance can be estimated. It illustrates that increasing *V*_G-MICRO_ (the microbial contribution) would increase total host heritability (Fig. A). It also suggests that when the microbiome is transmitted across generations, so is the genetic variation it harbors. However, the complex array of strategies driving the inheritance of the microbiome will affect transmission fidelity over generations. At one extreme, transmission fidelity could be very low (e.g., mostly environmentally acquired)—in which case parents and offspring could have different microbiomes. At the other extreme, transmission fidelity could be very high (e.g., strict vertical transmission), and the microbiomes of parents and offspring would be very similar. Transmission fidelity thus modulates the contribution of the microbiome from one generation to the next and therefore the host response to selection. In the context of host evolution, only the microbes that can shape host phenotypic variance and are also inherited thus contribute to the host response to selection. As transmission fidelity increases, then the contribution of *h*^2^_MICRO_ to total heritability increases, and so could the host response to selection (Fig. B). The effects of the microbiome on host phenotypic distribution will also affect the response to selection and the adaptive potential for the host population. Here, adaptive potential refers to factors favoring the ability of the population to respond to selection, primarily through the maintenance of additive genetic variance in the host and the microbiome^145^. When incorporating the microbial contribution to host phenotypes (i.e., *V*_G-MICRO_) the microbiome may also contribute to host total genetic variance and thus adaptive potential.
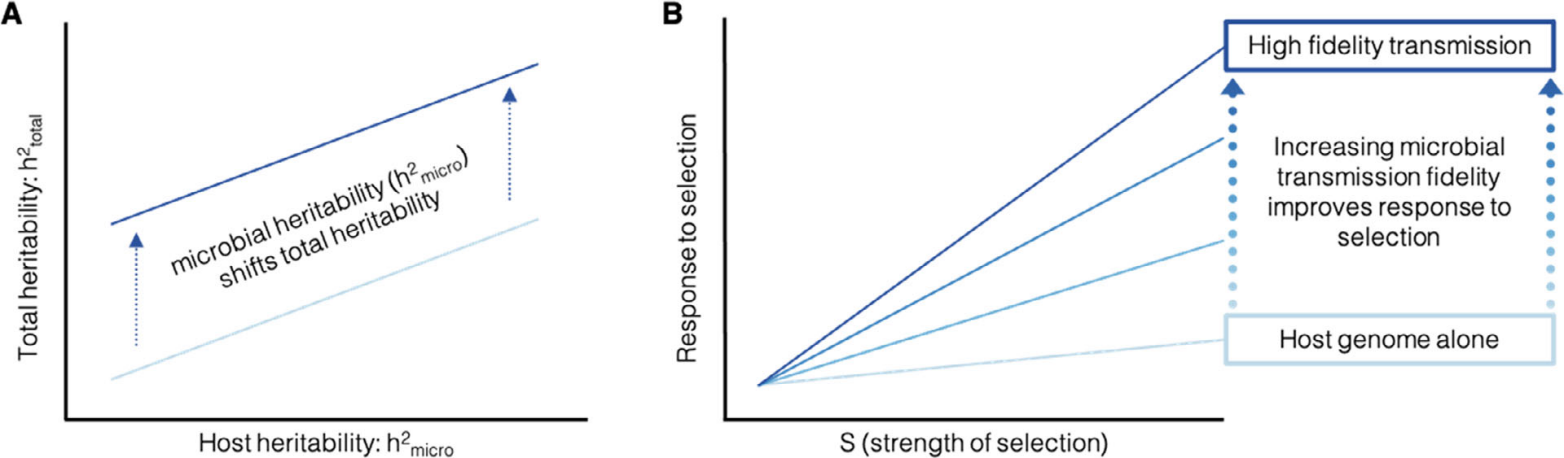

Conceptualized in Fig. C, we explore one hypothetical scenario where the microbiome shapes adaptive potential by contributing to total genetic variance across three different strengths of truncation selection. Blue and green distributions reflect the microbial contributions to host phenotypic distributions for mean and variance, respectively, while gray reflect the host distribution without microbiome. The vertical line represents the strength of selection, where the darker color reflects the trait values associated with individuals surviving selection. The percentages on the figure reflect the proportion of surviving individuals for each selection regime. The right most diagrams summarize how genetic variation shapes the response to selection and subsequently, adaptive potential for the three different scenarios. For weak selection, the effects of the microbiome on host phenotypic distribution have little impact as a broad range of trait values survive selection. However, under stronger selection, whether the microbiome shifts the phenotypic mean or variance of the host populations, the adaptive potential will change. Shifting the mean may increase the adaptive potential if the shifted distribution extends beyond the reach of selection. Similarly, increasing the variance may increase the adaptive potential for the population by increasing the proportion of individuals that survive selection (i.e., individuals harboring variable trait values). The hypothetical scenarios visualized in Fig. C are meant to illustrate how the microbiome could influence the adaptive potential of a host population. However, the latter will depend on many factors. For example, the host response will depend both on how interactions with the microbiome and environment shape phenotypic distributions, and this relationship may be non-linear. Incorporating more complex interactions including covariation between *G*_HOST_ × *G*_MICRO_ × *E* into total phenotypic variance is a key priority. Additionally, the microbial effects on adaptive potential assume a linear response as would be observed under truncation selection, which is likely uncommon in natural environments. More likely, the costs and benefits of microbiome-driven shifts in mean and variance will depend on the environmental variance^86,146^
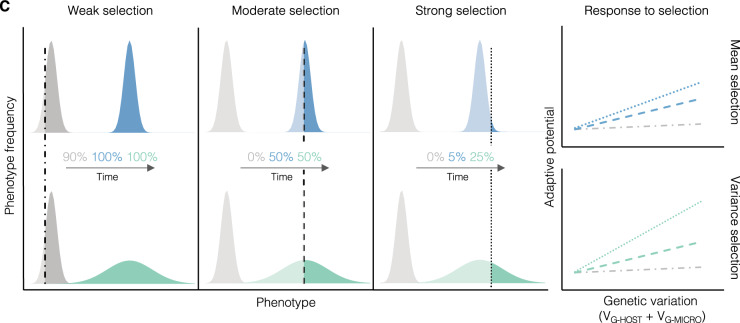

**Toward the derivation of formal quantitative genetic models that incorporate microbial variation:**

While we hope the framework we describe above provides a helpful starting point to study the host-microbiome interplay in evolution, it is an oversimplification. Formal derivation of quantitative genetic models that incorporates the contribution of the microbiome and distinguishes from host genetics still needs to be developed. Much of quantitative genetics relies on the assumptions that genotypes are fixed properties of an individual and that the transmission of genes to the next generation is unbiased (i.e., Mendelian genetics). As we outline above, the situation is more complicated with the microbiome, where *V*_G-MICRO_ may change over time and strict inheritance is not guaranteed. Tuning heritability by a transmission noise parameter may be an adequate solution to address this problem for some applications, as we suggested here. However, transmission may not be random in its effects on host phenotype. Transmission may also be influenced by the environment, and these host x environment interactions may lead to potential inflation of heritability estimates (this is an open problem). Other factors, such as microbe-microbe competition or conflict between host and microbiome are not presently incorporated^37^. Likewise, microbial demography (e.g., colonization and extinction), is not currently accounted for in this model, and variation in the microbiome over the host lifespan may actually be asymmetrical (having larger effects early in life^8,39^). Developing methods that account for temporal variation in the microbiome and its effects on host phenotypes remains challenging^147^.

A fast-growing body of data points to the dramatic influence of the microbiome on trait variation^1–9^. Ignoring or confounding the influence of *V*_G-MICRO_ with *V*_G-HOST_ seems increasingly inadequate. Other emerging fields, such as epigenetics, have benefited from this framework have developed theory building on quantitative genetics, despite potential challenges of the underlying assumptions^148–150^. Indeed, several recent papers have suggested the oversimplification of quantitative genetics has limited its utility, missing biologically important mechanisms that shape evolutionary dynamics like epigenetics, phenotypic plasticity, and co-evolution^125,151,152^. We raise these points to advocate for the development of theory and empirical approaches to better understand the complex relationship between host genetic variation, microbial variation, and heritability.

### A complex inheritance

Integrating microbial genetic variation into host evolutionary processes builds on ‘hologenome’ theory. The hologenome is defined as an evolutionary unit combining the eukaryotic host and all associated microbes^15,30,31^. Under this concept, evolution operates on this single unit because eukaryotic hosts are never isolated from microbes in the natural world^15,22^. Thus, the evolutionary fate of both hosts and microbes is intertwined. However, the intertwined fate of a single host-microbiome evolutionary unit is the focus of much criticism of the hologenome. One key criticism is that the host and microbiome rarely operate as one selective unit because transmission of the microbiome between host generations is rarely strictly vertical^6,7,9^. The microbiome can be transmitted via strict vertical transmission through embryos, but many microbes are also transmitted through quasi-vertical and environmental modes, like vaginal birth in mammals, regurgitation in birds, environmental inoculation in insects, or coprophagy in many taxa^32,33^. Environmental factors, like diet or climate, substantially influence the reservoir of possible microbial partners, leading to changes in the microbiome independent of any host evolution^34–36^. Host phenotypes also may be more strongly shaped by distinct microbial variation early in life, but because microbial dynamics operate at shorter timescales than host generations, those relevant microbes may not be faithfully transmitted across host generations^8,37–40^. Such environmental and demographic complexities muddle the inheritance of the microbiome, challenging our understanding of how and when the microbiome could influence host evolution^6–9,31^.

From a quantitative genetics perspective, heritability of the microbiome can be described as the proportion of microbial variation attributable to host genetic variance, just like any other complex trait^41^. Thus, heritable microbes are those where the relative abundance or community structure is associated with particular host genotypes^41,42^. Traditionally, only vertically transmitted microbes have been considered to be heritable, but the conflation of ‘microbial inheritance’ (i.e., transmission mode) and ‘microbial heritability’ (i.e., host genetic contribution to microbial variation) overlooks the many host systems that acquire their microbes through diverse transmission modes^43^. Indeed, in hosts dominated by environmentally acquired microbes, the contribution of host genetic variation to the relative abundance of particular microbes is as high as 42% in humans^44,45^, 39% in *Drosophila*^46^, and 25% in maize^47^. Interestingly, these and other studies show that not all components of the microbiome are heritable, with estimates ranging from 8 to b56% of microbes being transmitted faithfully^45,47–49^.

One might expect microbes with beneficial phenotypic effects to be more faithfully inherited. If a microbe contributes to a trait that promotes fitness in particular environments, then selection may reinforce faithful transmission to maintain the beneficial association, as observed in the many complex behaviors in host-microbe associations like aphid-*Buchnera*^50^, squid-*Vibrio*^51^, or beewolf-*Streptomyces* associations^52^. However, for many hosts, more complex communities are transmitted^53–55^. The drivers of this relationship between inheritance and phenotypic effects is not always clear. For example, in a UK twin study investigating the influence of host genetics on the microbiome, *Methanobrevibacter* species with the highest heritability had a strong association with low body-mass index^44,45^. However, another study in humans found low microbiome heritability, but microbial variation still explained 22-36% of metabolic traits^35^. Similarly, microbial variation explained 33% of weight gain in pigs^48^ and 13% of methane emissions in cows^49^ but also occurred largely independently of host genetic control of the microbiome. In other words, we still do not know whether the most heritable microbes explain the most significant variance in host traits.

Variation in transmission and the environment can impact the detection of heritable microbes. Controlling for transmission and environment are essential to identifying heritable microbes, and the disagreement between studies may result from differences in experimental design. The UK twin studies^44,45^ compared dizygotic and monozygotic twins to untangle maternal transmission and genetic contributions, while Rothschild et al.^35^ used large cohorts of unrelated individuals combined with metadata on diet and lifestyle to determine the environmental contribution. Animal and plant studies often use common garden experiments to expose different host genotypes to similar pools of available microbes^46–49^. The differences in these approaches may bias the ability to detect the contribution of heritable microbes to host trait variation, especially for hosts with largely environmentally acquired microbes. Despite these experimental limitations, these studies suggest that for a range of host traits, the microbiome contributes almost as much to phenotypic variance as do host genetics, even with the complicated inheritance of the microbiome.

Overall, these studies suggest that variation in how faithfully the microbiome is inherited may also influence host phenotypic variance. A quantitative genetics framework provides a path forward to partition the microbial contribution (V_G-MICRO_) to host phenotypes and allows us to further dissect the evolutionary consequences of this variation in transmission fidelity (Box 1). Through scaling the microbial effects by transmission fidelity, the microbiome could modulate the host response to selection. Microbes with phenotypic effects that are faithfully transmitted are likely to enhance the host phenotypic response to selection.

Our conceptualization suggests that the microbiome can modulate the host evolutionary potential, but there is currently limited empirical data to validate these conclusions. However, within the last 10 years, researchers have started to identify connections between the microbiome and host adaptation in diverse taxa and environments. We synthesized the literature and indeed found evidence suggestive of two common patterns in how the microbiome may shape host evolution connected to the processes suggested by our quantitative genetic framework: (1) hosts leverage locally adaptive microbes, shifting phenotypic means between host populations, and (2) the microbiome exposes novel host variation, changing host phenotypic variance within populations. These scenarios are not mutually exclusive, but as we explain below, may lead to different evolutionary trajectories for hosts.

### Hosts leverage locally adaptive microbes

Hosts may leverage microbes to acquire new traits that are adaptive in the local environment. Locally adaptive microbes may facilitate the ability of hosts to explore the fitness landscape to match local environmental stressors by shifting host phenotypic means (Fig. 1). In particular, microbes with larger effective population sizes, rapid generation time, and pangenomes may evolve novel functions faster than their hosts^56–59^, and hosts may also acquire these adaptive microbes from the standing microbial variation in the local environment. If hosts can leverage locally adaptive microbes, then hosts can increase survivorship and/or reproduction to rapidly adapt to novel environments.

There are a number of remarkable studies that illustrate the substantial impact of locally adaptive microbes on host phenotypes and fitness. For example, bean bugs can gain pesticide resistance by acquiring a pesticide degrading bacterium, *Burkholderia*^60–62^ that is already present in the soil environment. Many other hosts utilize their microbiome to detoxify harmful chemicals. In habitats with toxic creosote plants, woodrats have a gut microbiome that can degrade phenolic toxins; exposure to creosote resin predictably structures this microbiome, enriching for *Actinobacteria* that degrade phenols^63^ and enabling the woodrat to occupy a specialized dietary niche^63–67^. Woodrat populations naïve to creosote have distinct microbiomes that do not degrade phenolic toxins even after creosote resin exposure^68^; however, transfer of the toxin-adapted microbiome to these woodrats increases woodrat survival on a creosote diet^64^. The microbiome can also facilitate survival in other kinds of stressful environments. Plants on geothermal soils are associated with the thermotolerant endophyte *Curvularia*^69^; thermotolerant *Curvularia* can increase survival up to 40 °C in non-adapted tomatoes, while *Curvularia* isolated from non-geothermal soils did not^70^. Additionally, salt tolerant fungal endophytes can confer salt tolerance to non-adapted plant populations^70^. Overall, these examples show that hosts can utilize specific microbes with large phenotypic effects to specialize and persist in novel environments, likely adapting more rapidly than the host genome alone could.

### Microbial variation exposes novel host variation

During assembly of the microbiome, stochasticity and priority effects may create microbial variation between hosts within a population^8,38,71^. This change in microbial variation may increase phenotypic variability within populations (Fig. 1), enabling individuals to explore more phenotypic space^26,28^. Microbial variation would then enable the simultaneous exploration of different regions of fitness landscapes, changing the evolutionary trajectory of the host. We next review several examples that show how the microbiome increases phenotypic variance, but note that in natural populations, a combination of increases and decreases in phenotypic variance along with shifts in the mean likely occur.

Microbial variation increases host phenotypic variance in many organisms. For example, in *Drosophila*, different microbial communities increase the variance in larval development time, pupal weight, and adult weight compared between flies with different microbes and flies without microbes^72,73^. More so, different combinations of microbes shape life history traits in *Drosophila* nonlinearly, suggesting that microbial variation increases host phenotypic variance in complex ways^74^. Similarly, in *Daphnia*, different bacteria increase variance in body size and hatching success compared between sterile and among non-sterile treatments^75^. Microbiomes evolved for rapid flowering time in *Arabidopsis* increased variance in biomass compared to microbiomes evolved for slow flowering time^76^. In zebrafish, different bacteria species can either stimulate or suppress neutrophil responses, suggesting that variation in the microbiome is associated with varying immune responses to other bacteria^77^. In these cases, hosts are permissive to colonization by many microbes, and in turn, phenotypes respond to microbial variation. A major question for host-microbiome evolution is why some host phenotypes are responsive to diverse microbiomes while others maintain more specialized interactions?

The ability of the host to tune specific traits using diverse microbes may allow hosts to better match phenotypes to environmental stressors^16,17^, especially those stressors that may vary within populations. First, hosts that experience extreme variation in environment over their lifespan may need flexibility in their microbiome^16^. For example, coral microbiomes shift to protect against pathogens and extreme heat^78–80^. Bear microbiomes are enriched for microbes that harvest more energy during summer, most likely to ensure abundant fat accumulation before hibernation^81^. Second, the adaptive benefit of variable microbiomes may be especially important for organisms where high spatiotemporal variation in the environment occurs between parent and offspring. When offspring occupy different environments than parents, microbes may provide critical cues for local environments. For example, in *Daphnia*, microbes acquired from their local environment increased fecundity compared to those acquired via maternal transmission before diapause^75^. To best match phenotype to the local environment, hosts may frequently use microbial variation to alter developmental timing. Development may be especially responsive to microbial variation in many taxa, from insects to plants to crustaceans^82^. Developmental plasticity, through microbial variation, may expose novel phenotypic variation^83^, and this may in turn generate phenotypes that allow rapid adaptation to novel environments.

Particularly for organisms that live in fluctuating environments, the microbiome may enable a form of evolutionary bet-hedging. Bet-hedging occurs when phenotypic variation is maintained within a population to maximize long-term fitness of the lineage, despite shorter term fitness costs^84^. In the microbiome, bet-hedging could occur when hosts harbor associations with diverse microbes and responsive phenotypes. For some host systems, microbial variation may increase variability among individuals within a population, like when a diverse reservoir of microbes stochastically infects individuals, leading to increased inter-individual variation. Bet-hedging may also occur at the intra-individual scale, like when microbes have different phenotypic effects during different life stages. For example, studies have shown that *Lactobacillus* typically slows development in flies, suggesting different fitness costs in different environments^72,73^. However, on a nutrient poor diet, *Lactobacillus* increased in abundance and promoted larval growth^85^. These studies suggest the microbiome may enable a form of bet-hedging in *Drosophila* and potentially other hosts, but more work is necessary to explicitly test the role of the microbiome in bet-hedging^86^.

### Long-term effects of the microbiome on host evolution

So far, we have focused on how variation in the microbiome shapes both the mean and variance of host phenotypic traits. When the phenotypic distribution changes, then the response to selection in the host also changes (Box 1). Over time, the adaptive potential of the microbiome will also leave signatures of selection in the host genome as well. The role of the microbiome in long-term host evolution is poorly understood, but by drawing on studies in evolutionary ecology and traditional symbioses, we can draw basic predictions for the long-term effects of microbiome-induced phenotypic variation on host evolution.

Relying on locally adaptive microbes may facilitate host trait evolution in the short term—but what are the long-term evolutionary consequences of host reliance on locally adaptive microbes? If beneficial, we may expect that the frequency of those microbes will increase within the host population, and in turn, this evolutionary benefit may allow hosts to rapidly cross the fitness landscape. Thus, hosts should evolve to maintain locally adaptive microbes and their phenotypic effects to either host traits or mitigation of the environmental stressors, much like genetic accommodation or niche construction^83^. Host taxa then specialize in these unique niches, as observed for taxa like bark beetles^87^, pine weevils^88^, and coffee berry borers^89^ that use their microbiomes to detoxify plant secondary compounds in specialized niches.

When host taxa experience similar ecological processes, the microbiome may converge to provide similar functions, like conifer pests that use microbes to degrade otherwise toxic terpenes^87,88,90^. In all of these cases, selection may favor specific functions to ensure consistent benefits to these specialized host–microbe associations. However, it is unclear whether selection operates first on the standing pool of microbes in the environment (that are then acquired by hosts), or if selection on the host exerts selective pressure on the microbiome, which may in turn evolve novel traits, benefiting the host. In some cases, like for bean bugs, pesticide application first increases pesticide resistant *Burkholderia* in the soil, and then bean bugs acquire the pesticide resistance through *Burkholderia*^62^.

It is worth considering that when microbial variation exposes novel host phenotypic variation, hosts must deal with inconsistent benefits of the microbiome in the short-term. The benefits and stability of a given host-microbiome association will ultimately be a function of the environmental context, with different expectations for hosts that inhabit stable versus more rapidly fluctuating environments (as discussed in Bruijning et al.^86^).

However, relying on environmentally acquired microbes increases the probability of random microbial acquisition, like pathogens and non-adaptive microbes. If specific microbes are consistently beneficial, hosts should evolve mechanisms to ensure faithful microbial transmission, similar to traditional symbioses^91^. The quantitative genetic framework introduced above is focused on a specific functional trait, but this framework may also be extended to selection on transmission mechanisms as well. For example, the bean bug acquires pesticide-resistant *Burkholderia* even at only 0.04% of the total soil microbiome^62^. With a unique physical sorting structure in the midgut that imposes competition between different potential microbes, the bean bug ensures the establishment of only beneficial *Burkholderia* species^92,93^. Hosts can evolve mechanisms to either screen potential symbionts, like in the bean bug-*Burkholderia* example, or other behaviors that promote the acquisition of beneficial microbes, like coprophagy^32,33^. While it is still contentious if most microbiomes behave like traditional symbioses, ultimately, strict vertical transmission and extreme reliance on specialized microbes is likely an evolutionary dead end, constraining hosts and microbes to the ecological conditions that generated the symbiosis^94^. Furthermore, when hosts leverage locally adaptive microbes, different populations will have different locally adaptive associations, which will likely lead to increased genetic differentiation between host populations. Overall, the challenge is to determine how microbial genetic variation is linked with and influences host genetic change during adaptation. Integrating the microbiome into quantitative genetics provides a framework to generate formal predictions. However, as discussed next, accurately quantifying microbial variation and its effects on host phenotypic variation by taking advantage of clever experimental design are necessary to test these hypotheses.

### Experimental approaches to study the influence of the microbiome on host evolution

Above, we identified common scenarios of how the microbiome may influence host adaptation, with a focus on how host phenotypic distributions across individuals are shaped by microbial variation. With recent advances in sequencing technologies, microbial variation can be quantified at many different scales, from strains to whole community composition to complete ‘metagenomes’ (see Knight et al.^95^ for a recent review). A major challenge is to identify which scale of microbial variation (i.e., strain, community, metagenome) best measures microbial adaptation and influences host phenotypes^8,95,96^. Overcoming the technical hurdles in accurately quantifying microbial variation will be key to detecting how the microbiome influences the host evolutionary response.

The majority of host–microbiome interactions are characterized through “marker gene” studies (i.e., 16S rRNA for bacteria, ITS for fungi). Marker genes can classify microbial communities at broad taxonomic levels, are relatively inexpensive to sequence, and have established bioinformatic and analytical pipelines^95^. This approach demonstrates that microbial communities respond to many different kinds of selective pressures, like drought^97,98^, antibiotics^99,100^, different diets^101^, or warming environments^102^. Some studies show that microbial community diversity decreases in response to stressful environments^98–100^, while others show only shifts in community composition^97,101,102^. Marker gene studies are well-suited for taxonomically defining communities (though see^103^), but changes in microbial community composition do not always correlate to changes in host phenotype. For example, as measured by 16S rRNA marker genes, the macroalgae *Ulva* is colonized by different bacteria in different environments, but metagenomic data show the same core functional genes encoded by different bacterial taxa^104^. Similar patterns are observed in human microbiomes^105^ and the tree phyllosphere^106,107^. It remains unknown how taxonomic and functional identity provide different adaptive value for hosts. However, this conclusion may be strongly impacted by how microbial variation is characterized. Therefore, techniques beyond marker gene classification are needed to determine if and how microbial variation influences the host response to selection.

Strain-level analyses of microbial variation (i.e., polymorphisms and other genetic variants beyond marker genes) may provide more functional insight into the microbiome response to selection. For example, strain-level variation in honey bee microbes influences the response to pathogens or different diets for the hosts^108^. Strain-level analyses also provide insights into the stability of the microbiome across the lifetime of the host^54,109,110^ as well as transmission dynamics^111,112^. Metagenomes, which fully characterize the genes encoded by microbes, will provide crucial insights into functional variation of the microbiome^95,108,113,114^, like how bacterial metabolism varies across different niches within the same host^115^. This approach however remains costly, computationally intensive, and the results can be difficult to interpret. The emerging field of metatranscriptomics aims to study gene expression in the complex microbial communities^95,116,117^. Linking the metagenome to the metatranscriptome will be particularly insightful to understand how microbial variation influence host phenotypic variation^95,118^. For example, by comparing metagenomes to metatranscriptomes in the human gut, it was discovered that only a few specialized bacteria express unique biosynthesis pathways that may reflect the microbiome response to changing diets or other environmental perturbations in hosts^118,119^.

**Fig. 2:**
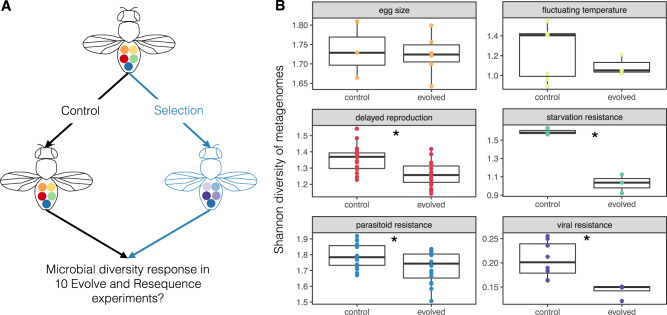
Microbiome responds to host experimental evolution. Experimental evolution is a powerful tool to understand the genetic basis of adaptation. **A** Hypothesized schematic of the microbial response during adaptation in flies. **B** Microbial diversity is frequently reduced in evolved populations during experimental evolution in flies. Data is reproduced from Henry & Ayroles^123^. Here, each point represents Shannon diversity of metagenomes from a pool of sequenced flies. Asterisks denote the experiments where microbial diversity is significantly different between control and evolved populations.

Experimental evolution can provide a powerful approach to investigate how microbial change interacts with host evolution^13,120,121^. In *Drosophila*, “Evolve and Resequence” (E&R) experiments, have uncovered many aspects of the genetic basis of adaptation to a diverse range of selective pressures^122^. E&R experiments use outbred populations, exert a selective pressure, and use sequencing to identify the genomic signatures of selection (Fig. 2). However, missing from this approach is whether and how the microbiome shapes the adaptive response of the host. As a first step in answering this question, the re-analysis of ten E&R experiments in *D. melanogaster*^123^ focusing on characterizing the microbial response to selection has been particularly insightful (Fig. 2). First, not all selective regimes had an effect on the microbiome. This observation is consistent with the idea that some phenotypes are more responsive to the influence of the microbiome than other phenotypes. Across the ten experiments, microbial diversity significantly decreased in four of the evolved populations (compared to the control populations), while it increased in one population. The decrease in diversity arises from a substantial increase in relative abundance for only a few bacteria, suggesting that flies are leveraging locally adaptive microbes. For example, for starvation resistance, *Wolbachia* increased in frequency following selection.

**Fig. 3:**
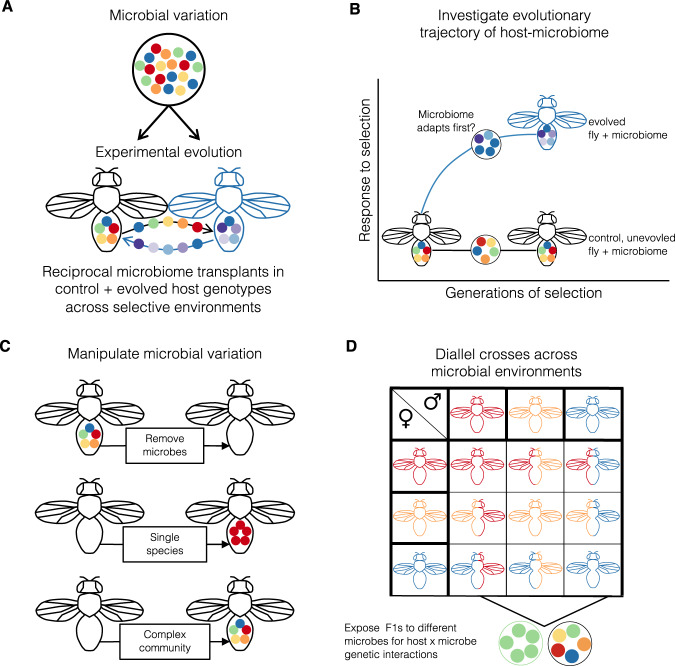
Partitioning microbial effects on host adaptation. Experimental approaches developed for variance partitioning in quantitative genetics can be a powerful way to assess the influence of the microbiome on host evolution. **A** Experimental evolution will provide critical insights into how hosts and microbiomes respond to stressful environments. By including the microbiome (visualized as different colored circles) in experimental evolution, then genetic responses in both host and microbiome can be measured following selection. At the end of experimental evolution, we expect both host and microbiome to adapt - visualized as blue fly and blue/purple microbes. Microbial evolution may occur at the strain level (e.g., when beneficial mutations in particular genes drive adaptation to the novel selective pressure). Alternatively, selection may increase the frequency of a particular microbial taxon, leading to loss of microbial taxa in the adapted microbiome. To test how the microbiome interacts with the host genome to influence host phenotypes, one can perform fully factorial, reciprocal transplants between host, microbiome, and environment. **B** Key insights will be gained from examining the evolutionary trajectory of alleles that emerge or change in frequency during experimental evolution. **C** To test how microbial variation influences host phenotype, hosts can be inoculated with different levels of microbial variation. Removing the microbiome through antibiotics (or other manipulations) will show how hosts respond to perturbation to their microbiomes. **D** Finally, diallel crosses can be used to show how host and microbial genetic variation interact. Diallel crosses are performed by crossing all possible combinations between inbred lines to each other in a common environment (represented by fly colors). Rearing F1s in different microbial environments will enable partitioning of the additive and nonlinear, epistatic components between host and microbial genetic variation.

While these results are correlative, this E&R survey suggests that the microbiome also responded to various selective regimes, and that microbiomes evolved in tandem with their hosts. However, additional work is necessary to understand if and how microbial evolution in these experiments also influenced host evolution. Like many other studies, we can only observe the endpoint of the host-microbiome evolutionary trajectory. Ultimately, understanding how the microbiome alters the evolutionary trajectory in hosts is the critical missing link^13,15,16,18,19,120,124–126^. Monitoring the temporal dynamics as populations respond to selection should provide the missing link—specifically, does the microbiome adapt faster than the host? And how frequently does the host utilize this rapid microbial evolution? A recent study, where the temporal evolutionary dynamics were monitored, suggests that environmentally acquired *Lactobacillus* bacteria in *Drosophila* evolve rapidly to nutrient-poor diets, and *Drosophila* can leverage rapid microbial adaptation independent of their own evolution^121^. We propose that experimental evolution, combined with statistical tools used in quantitative genetics, will be an important tool to understand how the microbiome shapes host phenotypic distributions, patterns of inheritance, and how these together influence host evolutionary trajectories (Fig. 3).

### A practical outlook

Natural variation in the microbiome may also provide new tools to combat a range of challenges with practical applications. Indeed, there are several promising avenues of research leveraging natural microbial variation in fields ranging from conservation biology to agriculture. For example, variants of *Pseudomonas* bacteria isolated from caves help protect vulnerable bats from the devastating fungal infections of white-nose syndrome^127,128^. A similar strategy is also in development to protect amphibians from chytrid pathogens^129^. From a public health perspective, manipulating the microbiome in disease vectors may reduce vector competence^130^. The success of cancer management can be influenced by the presence of particular gut microbes in humans and finding novel probiotics to improve the efficacy of chemotherapy is under development^131^. Isolating host-associated microbiomes from organisms in extreme environments, like geothermal soils^69,70^, will serve as reservoir of adaptive microbes for agriculture in changing environments^13,132^. Natural variation in the microbiome represents a largely untapped resource with many novel solutions to address applied challenges.

Our quantitative genetics framework provides a useful starting point that can be further developed to leverage the microbiome in applied challenges. For conservation efforts, ensuring that microbial diversity is buffered from anthropogenic effects may help endangered organisms persist and restore both free-living and within-host ecosystems^133–135^. The effect may be similar to the microbiome increasing phenotypic variation to buffer fluctuating environments. Second, probiotics could be used to increase host resistance to specific challenges^136^, much like when the microbiome shifts host phenotypic distributions. The evolutionary persistence of microbiome engineering will depend on how selection operates on both host and microbiome^13^. For this to work, our framework suggests that maintaining transmission fidelity will enhance host response to selection (Box 1). Finally, many of the studies cited here utilize highly controlled environment to test the microbial contribution to host phenotypes. Identifying the processes that shape host-microbiome interactions in the wild is a major missing link^135,137^. Understanding the match between host phenotypic effects with the environment will be key to leveraging the microbiome for applied challenges.

## Conclusions

In this perspective piece, we have identified scenarios of how the microbiome has the greatest potential to influence host evolution. Nevertheless, several key evolutionary questions remain to be addressed (see also Box 2): How does the microbiome change the host response to selection? What is the genetic basis of host responsiveness to microbial variation? Do the results of lab studies apply to microbiomes in the wild? Answering these questions will require a diversity of approaches using tools from quantitative genetics, community ecology, and genomics. Box 3

Several key analytical and theoretical gaps limit our current understanding of microbial influence on host evolution. More advanced bioinformatic tools are necessary to evaluate microbial diversity and its impact on host phenotypic diversity^95^. On the theoretical side, many aspects of the microbiome may poorly match the assumptions of theory developed for free-living organisms. Can we, for example, apply indirect selection^12^ or multilevel selection models (i.e., Price equation)^96^ to ask how independent or synergistic host and microbiome evolution are? One novel approach to detect the signatures of microbial variation shaping host evolution is ‘interspecies linkage disequilibrium’^138^, where selection may result in non-random allelic assortment of beneficial microbes and host genetic variation. The challenge is to identify how frequently variation in the microbiome increases host fitness, and whether this increases the probability of transmission across host generations.

Developing meaningful null models to describe host-microbiome evolution is an important and nontrivial challenge. A typical comparison would be between germ-free and conventionally reared hosts, where the null model would assume no difference. If hosts with a microbiome differed in their response to a selective pressure compared to the germ-free group, then the microbiome would be implicated in shaping host evolution. However, this may not be the most informative null, as how often have host organisms experienced a microbe-free world in their evolutionary history? Is the complete lack of a microbiome relevant to the evolutionary context in which organisms adapt to selective pressures? For some host taxa, recent work suggests that the modification and removal of the microbiome do not change host physiology^139,140^, and thus variation in the microbiome may not be evolutionary relevant for the host. First, we need more empirical data to calibrate the null model to understand how often the microbiome does not influence host phenotypes. Second, as outlined in Fig. 3, comparing evolved and ancestral microbiomes (and not only against germ-free conditions) across environments could greatly improve our understanding of host-microbiome evolution. Third, the null model should consider the balance in evolutionary interests between host and microbiome. We cannot assume that host-microbe associations evolve together to mutually benefit each partner^6^. Hosts and microbiomes will rarely have perfectly aligned evolutionary interests, suggesting a balance between cooperation and conflict^6,8,37^. Intergenomic conflict may maintain variation in microbial associations, but how often this influences host-microbiome evolution is not well understood^141^. Ultimately, the null hypothesis should drive appropriate experimental design to understand the balance between cooperation, conflict, and indifference between the host and microbiome during adaptation.

In conclusion, the contribution of the complex interplay between host and microbial genetic variation to host evolution is surprisingly understudied. Until recently, most quantitative and evolutionary genetic models addressing this topic have largely neglected the contribution of the microbiome to host phenotypic variance and evolution. Yet work from community ecology, quantitative genetics, and evolutionary biology suggest that the microbiome frequently shapes host phenotypic distributions across taxa and environments and may play a critical role in host evolution. Here, we have proposed a simple framework based on quantitative genetics that explicitly considers how genetic variation in the microbiome can extend the genetic repertoire of the host genome, influence host trait heritability, and subsequently impact host phenotypic evolution. Many challenges remain in this burgeoning field, but continuing advances in sequencing technology will facilitate the necessary characterization of host-microbiome evolutionary dynamics. Incorporating microbial variation into quantitative genetic models will provide fundamental novel insights into how selection operates across ecological and evolutionary scales.

Box 2: Key questions and research priorities *How does the microbiome change the host response to selection?* When the microbiome responds to selection, then does the microbiome reach a new adaptive peak before the host? Does the microbiome reinforce or dilute the selective forces acting on the host? What effect does the microbiome have on the distribution of host phenotypes within a population? Sequencing and microbiome transplantation along the course of experimental evolution will provide key insights into the role of microbiome in host adaptation. Equally important is investigating systems where microbes do not impact host adaptation^6,96,139^

*What is the genetic basis underlying host responses to microbial variation?* Are there microbes (or genes) with disproportionately large effects on host phenotype? Do few host genes enable specificity in microbial associations? What host genes integrate and transform microbial variation into phenotypic variation? To understand how host and microbial genomes interact, we will need to combine common marker gene sequencing approaches with new approaches in characterizing microbial genetic variation at the strain level^153–155^. Computational approaches that ask how variation in the microbiome interacts with host genetic variation, like interspecies linkage disequilibrium^138^ ­and interspecies eQTLs^156^, will illustrate mechanistic processes underlying microbiome-driven host adaptation.

*What ecological and evolutionary forces structure microbiomes in the wild?* What abiotic and biotic pressures influence microbial variation? Does the microbiome alter intra- and interspecific competitive dynamics among hosts? How faithfully are microbiomes transmitted across generations and/or environments? How stable are microbiomes over the lifespan of hosts? How does stability in the microbiome host shape phenotypic variation across environments? How frequently do microbiomes influence host fitness in the wild? To answer these questions, researchers should follow best practices to sample the microbiome in both hosts and the environment^95,137,157^. Identifying how the microbiome is transmitted and maintained in the wild will be crucial to understanding how the environmentally acquired microbiome shapes evolutionary processes.

Box 3: Glossary

1. Extended genotype: Hosts leverage genes encoded by the microbiome to extend the genetic repertoire and phenotypic variation, beyond the host genome alone^21^.
2. Extended phenotype: Organisms modify their local environment, extending the reach of the focal organism’s genes^11^.
3. Evolve and Resequence: Experimental evolution in diverse environments, then whole genome sequencing on pools of several replicated control and evolved populations. These experiments are designed to uncover genetic variants underlying adaptation^122^.
4. Hologenome/holobionts: The collection of host and all microbial genomes^14^. Holobiont refers to the combined organism and its microbiome as in individual, and selection acts upon the holobiont/hologenome as one collective unit^15^.
5. Interspecies linkage disequilibrium: Occurs when particular genetic variants in host are co-inherited with variants in the microbiome. Defined as *θ* by Wade^138^, if variants across species are mutually beneficial, selection will work to link together and maintain host and microbial variants, analogous to classical long-range linkage disequilibrium.
6. Marker genes: Genes used to classify taxonomic identity of microbes, like 16S rRNA for bacteria or ITS for fungi. Typically conserved within a microbial species, marker genes serve as common metric for microbial community diversity, but provide limited insight into functional variation^95^.
7. Metagenome: All genes encoded by the collective microbiome. The metagenome is characterized by whole genome sequencing and provides more functional insight than marker gene surveys^95,158^.
8. Microbial heritability: The proportion of variance in microbial composition due to host genetic factors^43^.
9. Microbial inheritance: The transmission mode of microbes, which does not necessarily depend on host genetic factors^43^.
10. Microbiome: The microbial community associated with a host. While many studies focus on bacteria, the microbiome also includes fungi, viruses, and other microbial eukaryotes that inhabit both internal and external habitats within the host^158^.
